# Association Between Long-Term Exposure to Particulate Matter and Glycated Hemoglobin Levels: A Cohort Study from the Korean Genome and Epidemiology Study

**DOI:** 10.3390/jcm15072797

**Published:** 2026-04-07

**Authors:** Kyeongmin Kwak, Saemi Jung, Daeil Kwon, Seryeon Lee

**Affiliations:** 1 Department of Occupational and Environmental Medicine, Korea University Ansan Hospital, Korea University College of Medicine, Ansan 15355, Republic of Korea; pathfinder81@korea.ac.kr; 2 Department of Occupational and Environmental Medicine, Centum General Hospital, Busan 48243, Republic of Korea; saemi.bright@gmail.com; 3 Buyeo-gun Public Health Center, Buyeo 33140, Republic of Korea; daeil90@gmail.com; 4 Department of Public Health, Korea University College of Medicine, Seoul 02841, Republic of Korea; 5 Division of Oncology and Hematology, Department of Internal Medicine, Korea University Ansan Hospital, Korea University College of Medicine, Ansan 15355, Republic of Korea

**Keywords:** air pollution, particulate matter, glycated hemoglobin, HbA1c, glucose metabolism, effect modification, KoGES, cohort study

## Abstract

**Background:** Ambient air pollution, particularly particulate matter (PM), has been linked to metabolic disorders, including diabetes. We evaluated associations between long-term exposure to coarse particulate matter (PM_10_) and fine particulate matter (PM_2.5_) and glycated hemoglobin (HbA1c) levels in a Korean population and assessed whether specific subgroups exhibited heightened susceptibility. **Methods:** We analyzed 6940 participants without diabetes from the Korean Genome and Epidemiology Study (KoGES) Ansan-Ansung cohort. Participants contributed 35,395 observations across a mean follow-up of 5.1 visits (2005–2017). Linear mixed models estimated associations between PM exposure and HbA1c while adjusting for covariates, including body mass index (BMI), time, and region. Subgroup analyses stratified by sex, age, BMI, region, education, smoking status, drinking status, and exercise. **Results:** Higher long-term PM_10_ exposure was associated with elevated HbA1c (β = 0.0347 per interquartile range [IQR] increase of 9.48 μg/m^3^; 95% CI: 0.0220, 0.0473; *p* < 0.001). PM_2.5_ showed a comparable positive association (β = 0.0166 per IQR of 8.67 μg/m^3^; 95% CI: 0.0010, 0.0321; *p* = 0.037). Associations were stronger among older adults (≥60 years: β = 0.0789 vs. <60 years: β = 0.0210; *p*-interaction < 0.001), rural Ansung residents (β = 0.0963 vs. Ansan: β = 0.0398; *p*-interaction < 0.001), participants with lower educational attainment (≤middle school: β = 0.0637; *p*-interaction < 0.001), and never smokers (β = 0.0455; *p*-interaction = 0.035). **Conclusions:** Among nondiabetic Korean adults, long-term PM_10_ exposure was associated with higher HbA1c; PM_2.5_ demonstrated a similar positive association. Associations were more pronounced among older adults, rural residents, individuals with lower educational attainment, and never smokers. These findings support ambient air pollution as an environmental contributor to subclinical glycemic changes and underscore the need for targeted public health strategies for vulnerable populations.

## 1. Introduction

Diabetes prevalence has risen sharply worldwide over recent decades, creating a substantial global disease burden. The International Diabetes Federation estimated that 537 million adults were living with diabetes in 2021 and projected this number to reach 783 million by 2045 [1]. Type 2 diabetes mellitus (T2DM) accounts for more than 90% of cases and is typically attributed to genetic predisposition, obesity, physical inactivity, and dietary factors [2]. Growing evidence, however, indicates that environmental exposures—particularly ambient air pollution—may constitute an additional, increasingly important determinant of diabetes development and progression [3,4].

The Global Burden of Disease Study 2019 [5] provided robust evidence linking air pollution to diabetes. The analysis estimated that approximately one-fifth of the global T2DM burden is attributable to fine particulate matter (PM_2.5_) exposure. It also reported 3.78 deaths per 100,000 population and 167 disability-adjusted life-years (DALYs) per 100,000 population attributable to PM_2.5_ [5]. Since 1990, this attributable burden has increased by approximately 50%, driven largely by population growth and aging—particularly in Asia, sub-Saharan Africa, and South America [5,6]. These findings underscore the need to characterize air pollution–diabetes relationships across diverse populations. Biological evidence also supports this link: PM_2.5_ exposure can trigger systemic oxidative stress, inflammation, endothelial dysfunction, and mitochondrial dysfunction, all of which may disrupt glucose metabolism [7,8,9,10,11,12].

Epidemiological evidence has expanded in parallel. A meta-analysis of cohort studies reported that each 10 μg/m^3^ increase in long-term PM_2.5_ exposure is associated with a 25% higher T2DM risk (relative risk [RR] 1.25; 95% confidence interval [CI]: 1.10–1.43) [13]. Large-scale cohort studies in diverse populations have consistently linked long-term PM_2.5_ exposure with elevated diabetes risk [14,15,16]. Most longitudinal studies, however, have treated diabetes incidence as a binary outcome. Evaluating continuous glycemic markers in nondiabetic populations may more sensitively capture subclinical metabolic changes associated with air pollution. Glycated hemoglobin (HbA1c), which reflects average blood glucose over the preceding 2–3 months, is well suited to this purpose. Unlike binary diabetes diagnosis, continuous HbA1c measurement can detect gradual subclinical glycemic shifts, providing a sensitive marker for early metabolic effects of environmental exposures before overt disease develops.

In South Korea, PM_2.5_ concentrations remain higher than those in North America and Western Europe [17]. Although levels have declined [18], they still substantially exceed the World Health Organization (WHO) annual guideline of 5 μg/m^3^ [19]. Studies using Korea National Health and Nutrition Examination Survey (KNHANES) data have linked short- and mid-term air pollution exposure to changes in fasting blood glucose and HbA1c [20]. Longitudinal cohort studies evaluating air pollution in relation to glycemic markers in Korean populations, however, remain limited—particularly studies assessing effect modification by age, region, and lifestyle factors.

To address this gap, we used the Korean Genome and Epidemiology Study (KoGES), a large longitudinal cohort initiated in 2001 that links 1 km-resolution air pollution estimates to participants’ residential addresses [21,22]. We examined associations between long-term particulate matter (PM) exposure and HbA1c levels after adjusting for temporal trends and evaluated whether demographic, lifestyle, and regional factors modified these associations.

## 2. Materials and Methods

### 2.1. Study Population

We analyzed data from the Korean Genome and Epidemiology Study (KoGES) Ansan–Ansung cohort, a prospective community-based cohort established in 2001 [21]. This cohort includes adults aged 40–69 years residing in the urban Ansan and rural Ansung areas of Gyeonggi Province, South Korea.

**Figure 1:**
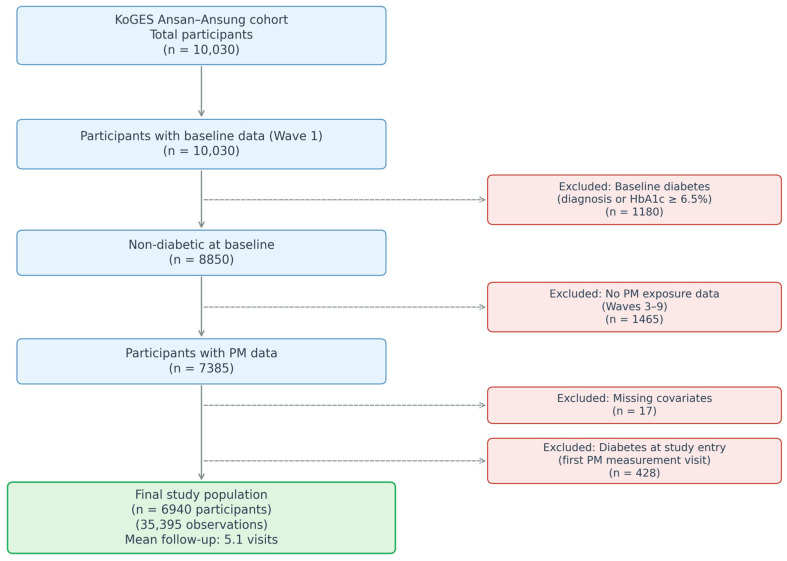
Flow diagram of participant selection from the Korean Genome and Epidemiology Study (KoGES) Ansan–Ansung cohort.

Of the 10,030 participants enrolled at baseline (Wave 1, 2001–2002), we excluded 1180 with prevalent diabetes (defined as physician-diagnosed diabetes or HbA1c ≥ 6.5%). From the remaining 8850 participants, we excluded 1465 who lacked PM exposure estimates during follow-up (Waves 3–9, 2005–2017) because modeled air pollution data were available only from 2005 onward. Among the 7385 participants with PM data, we further excluded 428 who developed diabetes before their first visit with PM measurement (study entry) and 17 with missing covariates (including body mass index [BMI]). The final analytical sample comprised 6940 participants who contributed 35,395 observations across a mean of 5.1 visits (Figure 1).

### 2.2. Exposure Assessment

We estimated ambient coarse particulate matter (PM_10_) and PM_2.5_ concentrations using Community Multiscale Air Quality (CMAQ) modeling. This approach integrates meteorological data, emission inventories, and atmospheric chemistry to generate spatially resolved pollution fields, calibrated against ground-level monitoring station measurements [22]. The model provided estimates at 1 km resolution. For each participant, we calculated 1-year average concentrations before each examination visit based on residential address. As a sensitivity analysis, we calculated 1-month, 3-month, and 6-month average concentrations to examine exposure–response gradients across temporal windows. The 1-week exposure analysis was exploratory—designed to assess whether short-term fluctuations were reflected in HbA1c levels—recognizing that HbA1c primarily reflects glycemic status over the preceding 2–3 months.

### 2.3. Outcome Measurement

The primary outcome was HbA1c, measured at each follow-up visit. As a well-established indicator of glycemic control, HbA1c reflects mean blood glucose over the previous 2–3 months. Measurements were obtained by high-performance liquid chromatography using the Bio-Rad VARIANT II system (Bio-Rad Laboratories, Hercules, CA, USA) [23], which is certified by the National Glycohemoglobin Standardization Program (NGSP) and traceable to the Diabetes Control and Complications Trial (DCCT) reference method [24]. All measurements were performed in centralized laboratories following standardized protocols [21].

### 2.4. Covariates

Time-fixed covariates included sex (male/female), educational attainment (≤middle school, high school, ≥college), and region (Ansan/Ansung). Time-varying covariates included age, BMI, smoking status (never/former/current smoker), drinking status (never/former/current drinker), and regular exercise (yes/no). We included study visit (Waves 3–9) to adjust for temporal trends. Linear mixed models accommodate unbalanced data by design; participants contributed all available observations without imputation of missing visits.

### 2.5. Statistical Analysis

We used linear mixed models (LMMs) to evaluate associations between PM exposure and HbA1c levels while accounting for repeated measurements within individuals. Each model included a participant-specific random intercept and fixed effects for PM exposure, all covariates listed in Section 2.4, and study visit. Time-varying covariates were updated at each visit. PM concentrations were scaled by their interquartile range (IQR) to improve interpretability.

We fitted separate models for each PM metric: PM_10_ and PM_2.5_. Subgroup analyses were conducted by sex, age (<60 vs. ≥60 years), BMI (<25 vs. ≥25 kg/m^2^), region (Ansan vs. Ansung), educational attainment (≤middle school, high school, ≥college), smoking status (never/former/current smoker), drinking status (never/former/current drinker), and regular exercise (yes/no). We did not include month-specific indicators, temperature, or humidity as separate covariates because study visit (Waves 3–9, categorical) captured broad temporal trends including seasonal patterns, and HbA1c integrates glycemic status over 2–3 months, substantially attenuating short-term meteorological influences. Effect modification was assessed by adding interaction terms between PM exposure and each subgroup variable. *p*-values for interaction were calculated using Wald tests. All analyses were conducted in R software version 4.4.1 using the lme4 and lmerTest packages. To assess multicollinearity between PM exposure and study visit, we calculated generalized variance inflation factors (GVIF). We also examined pairwise Pearson correlations among visit number, PM concentrations, and HbA1c to characterize the temporal correlation structure.

## 3. Results

### 3.1. Baseline Characteristics

Table 1 summarizes baseline characteristics for the 6940 participants at their first observation. Mean age was 56.88 years (standard deviation [SD] 9.03), and 53.2% of participants were female. Mean BMI was 24.33 kg/m^2^ (SD 3.07); 38.8% were classified as obese (BMI ≥ 25 kg/m^2^). Most participants (63.0%) were never smokers, whereas 18.8% were former smokers and 18.2% were current smokers. For alcohol consumption, 48.1% were never drinkers and 47.0% were current drinkers. Regular exercise was reported by 36.5% of participants. Mean HbA1c was 5.44% (SD 0.38). Mean 1-year concentrations were 64.47 μg/m^3^ (SD 4.73) for PM_10_ and 32.68 μg/m^3^ (SD 3.13) for PM_2.5_. Across all observations, 1-year PM_10_ concentrations ranged from 29.36 to 138.63 μg/m^3^ and PM_2.5_ from 14.44 to 66.81 μg/m^3^.

Demographic and lifestyle characteristics differed significantly between regions. Compared with Ansan participants, Ansung participants were older, had lower educational attainment, were less likely to exercise regularly, and had slightly higher HbA1c levels (all *p* < 0.001). BMI was similar between regions (*p* = 0.244).

### 3.2. Temporal Trends in PM Exposure and HbA1c

Table 2 summarizes temporal changes in PM exposure and HbA1c levels across study visits. PM concentrations declined markedly over the study period. Mean 1-year PM_10_ exposure fell from 66.85 μg/m^3^ in Wave 3 (2005–2006) to 50.56 μg/m^3^ in Wave 9 (2017–2018)—a 24% reduction. Similarly, 1-year PM_2.5_ exposure decreased from 33.21 μg/m^3^ to 25.16 μg/m^3^ (24% reduction). In contrast, HbA1c rose steadily over this interval, increasing from 5.37% to 5.76%.

### 3.3. Association Between PM Exposure and HbA1c

Table 3 summarizes results from linear mixed models evaluating long-term PM exposure. After adjustment for sex, education, region, age, BMI, smoking, drinking, exercise, and study visit, 1-year exposures to PM_10_ and PM_2.5_ were significantly associated with higher HbA1c levels. Each IQR increase in PM_10_ (9.48 μg/m^3^) corresponded to a 0.0347 percentage-point increase in HbA1c (95% CI: 0.0220, 0.0473; *p* < 0.001). The association for PM_2.5_ was also positive but smaller (β = 0.0166 per IQR of 8.67 μg/m^3^; 95% CI: 0.0010, 0.0321; *p* = 0.037). Among covariates, female sex, older age, higher BMI, and smoking were associated with elevated HbA1c, whereas current drinking and regular exercise were associated with lower HbA1c. Study visit showed a strong positive association: HbA1c increased progressively from Wave 3 through Wave 9.

Short-term exposures exhibited different patterns across exposure windows. For PM_10_, associations were inverse at the 1-week (β = −0.0094) and 1-month windows (β = −0.0022), became positive at 3 months (β = 0.0074) and 6 months (β = 0.0056), and reached the strongest positive estimate at 1 year (β = 0.0347, *p* < 0.001). For PM_2.5_, associations remained null or inverse at shorter windows and became positive only at the 1-year window (β = 0.0166, *p* = 0.037). Full results across all windows appear in Supplementary Table S1. Generalized variance inflation factors confirmed acceptable multicollinearity levels between PM exposure and study visit for both models (all adjusted GVIF < 2.5) (Supplementary Table S2).

### 3.4. Subgroup Analysis

Table 4 summarizes subgroup analyses of long-term (1-year) PM exposure. Age, BMI, region, education, and smoking status significantly modified the associations for both PM_10_ and PM_2.5_ (all *p*-interaction < 0.05). In contrast, sex, drinking status, and exercise showed no significant interactions.

Across both pollutants, associations were consistently stronger among older adults (≥60 years), Ansung (rural) residents, participants with lower educational attainment, and never smokers. For PM_10_, older adults exhibited an association approximately fourfold stronger than younger participants (β = 0.0789; 95% CI: 0.0549, 0.1028 vs. β = 0.0210; 95% CI: 0.0060, 0.0361; *p*-interaction < 0.001). Similarly, Ansung residents demonstrated a 2.4-fold greater association than Ansan residents (β = 0.0963; 95% CI: 0.0733, 0.1194 vs. β = 0.0398; 95% CI: 0.0228, 0.0568; *p*-interaction < 0.001). A clear educational gradient emerged: associations were strongest among participants with middle-school education or below (PM_10_: β = 0.0637; 95% CI: 0.0447, 0.0827; PM_2.5_: β = 0.0502; 95% CI: 0.0272, 0.0732), whereas college graduates showed no significant association (both *p*-interaction < 0.001). Significant associations appeared only among never smokers for PM_10_ (β = 0.0455; 95% CI: 0.0298, 0.0613; *p*-interaction = 0.035) and PM_2.5_ (β = 0.0326; 95% CI: 0.0132, 0.0520; *p*-interaction = 0.046). Although point estimates were higher among women, non-obese participants, and never drinkers, interactions by sex, drinking status, and exercise did not reach statistical significance for either pollutant.

## 4. Discussion

In this longitudinal study of 6940 Korean adults without diabetes, higher long-term PM_10_ exposure was significantly associated with elevated HbA1c levels. PM_2.5_ demonstrated a similar positive association. We also identified clear effect modification by age, BMI, region, education, and smoking status. Associations were stronger among older adults, rural residents, participants with lower educational attainment, and never smokers; suggestive but nonsignificant differences emerged by sex. These findings reinforce the evidence linking air pollution to metabolic health and underscore the need to identify—and protect—populations at heightened risk.

The estimated effect sizes warrant careful clinical interpretation. A 0.0347 percentage-point increase in HbA1c per IQR increase in PM_10_ may appear modest at the individual level. At the population scale, however, even small shifts in the HbA1c distribution can push more individuals across clinically meaningful thresholds, such as the transition from normoglycemia to prediabetes (HbA1c ≥ 5.7%) [25]. Furthermore, the effect sizes we observed are comparable to those reported for other modifiable risk factors in population-based studies. The substantially larger effects in susceptible subgroups—for example, β = 0.0963 in rural residents and β = 0.0789 in older adults for PM_10_—suggest clinically relevant individual-level impacts in these populations.

The positive association between long-term PM exposure and HbA1c aligns with prior epidemiologic evidence. A KNHANES study found that mid-term PM_2.5_ was associated with a 0.07 percentage-point increase in HbA1c per IQR increment (60-day moving average) in Korean adults, with substantially larger effects in diabetic males aged ≥65 years [20]. Similar associations appear across populations: older Americans showed a 1.8% increase in HbA1c per IQR of PM_2.5_ among those with diabetes [26], while a German cohort documented a 0.07 percentage-point increase in those without diabetes [27]. Beyond glycemic markers, large cohort studies consistently link long-term PM_2.5_ to diabetes incidence. Among 1.7 million U.S. veterans (median follow-up 8.5 years), PM_2.5_ was significantly associated with increased diabetes risk [14]. In a Taiwanese retrospective cohort of 158,038 individuals, those in the highest PM_2.5_ quartile had 42% higher diabetes risk than the lowest (HR 1.42, 95% CI: 1.32–1.53) [15]. These findings underscore the importance of accounting for temporal trends in long-term exposure studies.

The estimated effect sizes (0.0347 percentage points for PM_10_ and 0.0166 percentage points for PM_2.5_ per IQR increase) may seem small for an individual. Air pollution exposure, however, is widespread and largely involuntary; even modest HbA1c shifts can translate into substantial population-level burden [28]. Notably, effects were much larger in susceptible subgroups for both pollutants. For PM_10_, β increased to 0.0789 in older adults and 0.0963 in rural residents. PM_2.5_ showed similarly elevated estimates (β = 0.0692 and β = 0.0894, respectively)—effect sizes more likely to influence individual-level glycemic progression. These associations persisted after adjusting for BMI as a time-varying covariate, suggesting that differences in adiposity do not fully explain the PM–HbA1c relationship. We also detected effect modification by BMI for both pollutants (both *p*-interaction < 0.001). Associations were stronger in the nonobese group (PM_10_: β = 0.0377; PM_2.5_: β = 0.0190) than in the obese group (PM_10_: β = 0.0264; PM_2.5_: β = 0.0027, nonsignificant). This pattern suggests greater susceptibility among lean individuals [29], potentially because obesity is already characterized by elevated oxidative stress and insulin resistance [30,31], which may partially overlap with PM-driven metabolic pathways.

Sex-stratified analyses revealed significant PM–HbA1c associations in women (PM_10_: β = 0.0537; PM_2.5_: β = 0.0474) but not in men (PM_10_: β = 0.0103; PM_2.5_: β = −0.0206). However, interaction terms were not statistically significant (PM_10_: *p*-interaction = 0.339; PM_2.5_: *p*-interaction = 0.588), indicating that the apparent sex difference was not formally confirmed. Canadian [32] and Chinese [33] cohorts similarly documented larger effect estimates in women despite non-significant sex interactions. These findings suggest that apparent sex differences may reflect sampling variability rather than true effect modification.

Age-stratified analyses revealed stronger associations in older adults (≥60 years; PM_10_: β = 0.0789; PM_2.5_: β = 0.0692) than in younger participants, with significant effect modification (both *p*-interaction < 0.001). This age gradient aligns with evidence that susceptibility to air pollution increases with aging. A Chinese cross-sectional study likewise reported stronger PM_2.5_–diabetes associations in older adults (≥65 years), with significant age-based effect modification (*p*-interaction = 0.03) [34]. Several mechanisms may plausibly contribute to this vulnerability: age-related declines in antioxidant defense and oxidative stress responses [35], greater cumulative exposure across the life course, a higher burden of comorbidities that may potentiate PM effects, and reduced physiologic reserve in older individuals [36].

The stronger PM–HbA1c associations in rural Ansung than urban Ansan likely involve factors beyond PM concentrations alone, including differences in PM composition, population characteristics, and lifestyle patterns. A parallel finding in 20 million Chinese women showed stronger PM_2.5_–fasting blood glucose associations in rural versus urban populations [16]. These contrasts may partly reflect differences in particle composition: rural areas experience greater agricultural emissions and biomass burning [37], generating organic matter and black carbon components linked to metabolic effects [38]. Behavioral factors may further increase effective exposure: rural residents often spend more time outdoors and may have fewer means of limiting exposure during high-pollution periods [39].

We also observed a pronounced educational gradient in the PM–HbA1c association. Effects were strongest among participants with middle school education or less (PM_10_: β = 0.0637; PM_2.5_: β = 0.0502), whereas college graduates showed no significant association (both *p*-interaction < 0.001). The consistency of this pattern across PM_10_ and PM_2.5_ suggests that socioeconomic disadvantage, proxied by educational attainment, may heighten susceptibility to air pollution–related metabolic effects [40]. Lower educational attainment is associated with residence closer to pollution sources, poorer access to healthcare and preventive resources, and fewer opportunities for protective behaviors (e.g., indoor air filtration or private transportation) [40,41]. From an environmental justice perspective, these findings reinforce that socioeconomically disadvantaged groups bear a disproportionate share of air pollution–related health burden [42], underscoring the need for equity-focused air quality policies and targeted public health interventions.

The finding that statistically significant associations appeared only among never smokers—and were weaker among former and current smokers—may seem counterintuitive. However, similar patterns have been reported previously. In a Danish cohort, associations between air pollution and incident diabetes were strongest among nonsmokers [43]. One plausible explanation is that tobacco smoke and ambient PM share combustion-derived chemical constituents and activate overlapping oxidative stress and inflammatory pathways [44]. Among smokers, a higher baseline oxidative and inflammatory burden may blunt the additional impact of ambient PM exposure.

The exposure–response gradient across temporal windows supports the biological plausibility of our primary findings. For PM_10_, associations transitioned from inverse at shorter windows (1 week, 1 month) to positive at 3 months and beyond, consistent with the 2–3-month integration period of HbA1c. For PM_2.5_, positive associations emerged only at the 1-year window, suggesting that fine particle effects on glycemic metabolism may require more sustained exposure to become detectable. The inverse associations at shorter windows likely reflect seasonal confounding rather than true protective effects: PM concentrations in Korea peak during colder months when indoor time increases, reducing effective personal exposure even as ambient levels rise. Residual confounding from unmeasured time-varying behaviors may also contribute. Prior studies have likewise reported null associations for short-term PM exposure and HbA1c, with significant associations observed primarily for longer exposure windows that align with the biomarker’s time course [45]. Together, these findings underscore the importance of matching the exposure assessment window to the outcome’s biological time course.

PM exposure alters glucose metabolism through multiple mechanisms. PM induces systemic oxidative stress and inflammation, which promote insulin resistance by activating inflammatory cytokines, impairing insulin signaling, and disrupting adipose tissue function [7,8]. These pathways also mediate PM-related cardiovascular disease [46,47]. Because of its small aerodynamic diameter (≤2.5 μm), PM2.5 penetrates the respiratory tract and enters the bloodstream, causing endothelial and mitochondrial dysfunction [9,10]. Supporting these observations, animal studies demonstrate that PM2.5 directly induces glucose intolerance, visceral inflammation, and insulin resistance [11,12]. PM attenuates key insulin signaling mediators, including the IRS-1/AKT and PI3K/AKT pathways [48,49].

This study has several strengths. First, we used the well-established KoGES cohort and leveraged repeated measurements across a long follow-up period. Second, we explicitly addressed temporal confounding: PM concentrations declined by approximately 24% over the study period, whereas HbA1c increased as participants aged. Without time adjustment, these opposing trends can generate spurious inverse associations. After including study visit as a covariate, the expected positive association emerged, emphasizing the importance of accounting for temporal trends in longitudinal air pollution research. Third, comprehensive subgroup analyses identified potentially vulnerable groups, including women, older adults, and rural residents. Fourth, high-resolution (1 km) air pollution estimates likely improved exposure assignment. Fifth, reverse causality is unlikely in this context because all participants were nondiabetic at baseline, HbA1c levels in the nondiabetic range are unlikely to influence ambient PM concentrations or residential location choices, and participants were censored at diabetes diagnosis.

We note several limitations. First, the study population was limited to two Korean regions, potentially restricting generalizability. Although Ansan and Ansung represent distinct rural and urban settings, they may not fully capture regional diversity in PM composition and exposure patterns. Generalizability to other countries with different pollution sources remains uncertain. Second, lack of data on indoor air pollution and individual exposure behaviors may have resulted in exposure misclassification. Third, unmeasured factors—such as dietary habits or genetic susceptibility—may have confounded results despite covariate adjustment. Fourth, we could not identify which PM components most strongly influenced HbA1c. Fifth, despite standardized NGSP-certified HPLC measurement (Bio-Rad VARIANT II) in centralized laboratories, minor measurement variability across the 12-year follow-up cannot be fully excluded. However, including study visits as a categorical covariate in all models would absorb any systematic wave-level differences in assay performance. Sixth, HbA1c may be influenced by non-glycemic factors such as iron deficiency anemia, hemolytic conditions, or hemoglobin variants, which were not systematically assessed in this study.

From a clinical and public health perspective, these findings indicate that ambient air pollution may contribute to glycemic changes, particularly among nondiabetic individuals susceptible to metabolic deterioration. Although the estimated effect sizes were modest, the population impact could be substantial because PM exposure is widespread. The WHO recently updated its air quality guidelines, recommending annual mean PM_10_ concentrations below 15 μg/m^3^ and PM_2.5_ below 5 μg/m^3^ [19]. PM concentrations in our study exceeded these targets, highlighting the need for continued air quality improvements. Given the stronger associations observed among older adults, rural residents, and participants with lower educational attainment, public health interventions should prioritize these higher-risk groups.

## 5. Conclusions

This longitudinal cohort study demonstrates that prolonged PM_10_ exposure is significantly associated with elevated HbA1c levels in nondiabetic Korean adults; PM_2.5_ exhibited a similar positive trend. These associations were more pronounced among older adults (≥60 years), rural residents, individuals with lower educational attainment, and never smokers. Our findings support the recognition of ambient air pollution as an environmental contributor to subclinical glycemic alterations and highlight the importance of targeted public health interventions for susceptible populations. Future research should elucidate the biological mechanisms underlying these differential vulnerabilities.

## Figures and Tables

**Table 1:** Baseline characteristics of study participants at first observation.

Variable	Category/Summary	Overall (*n* = 6940)	Ansan (*n* = 3435)	Ansung (*n* = 3505)	*p* Value
Age (years)	Mean (SD)	56.88 (9.03)	53.31 (7.41)	60.38 (9.12)	<0.001
Sex, *n* (%)	Male	3250 (46.8)	1730 (50.4)	1520 (43.4)	<0.001
	Female	3690 (53.2)	1705 (49.6)	1985 (56.6)	
BMI (kg/m^2^)	Mean (SD)	24.33 (3.07)	24.37 (2.79)	24.28 (3.33)	0.244
BMI category, *n* (%)	<18.5	145 (2.1)	36 (1.0)	109 (3.1)	<0.001
	18.5–22.9	2213 (31.9)	1067 (31.1)	1146 (32.7)	
	23.0–24.9	1888 (27.2)	1011 (29.4)	877 (25.0)	
	≥25.0	2694 (38.8)	1321 (38.5)	1373 (39.2)	
Education, *n* (%)	≤Middle school	3744 (53.9)	1133 (33.0)	2611 (74.5)	<0.001
	High school	2195 (31.6)	1553 (45.2)	642 (18.3)	
	≥College	1001 (14.4)	749 (21.8)	252 (7.2)	
Smoking status, *n* (%)	Never	4372 (63.0)	2075 (60.4)	2297 (65.5)	<0.001
	Former	1306 (18.8)	760 (22.1)	546 (15.6)	
	Current	1262 (18.2)	600 (17.5)	662 (18.9)	
Alcohol consumption, *n* (%)	Never	3341 (48.1)	1532 (44.6)	1809 (51.6)	<0.001
	Former	334 (4.8)	152 (4.4)	182 (5.2)	
	Current	3265 (47.0)	1751 (51.0)	1514 (43.2)	
Regular exercise, *n* (%)	No	4404 (63.5)	1767 (51.4)	2637 (75.2)	<0.001
	Yes	2536 (36.5)	1668 (48.6)	868 (24.8)	
HbA1c (%)	Mean (SD)	5.44 (0.38)	5.40 (0.37)	5.49 (0.38)	<0.001
PM_10_, 1-year (µg/m^3^)	Mean (SD)	64.47 (4.73)	63.06 (4.47)	65.86 (4.57)	<0.001
PM_2.5_, 1-year (µg/m^3^)	Mean (SD)	32.68 (3.13)	30.99 (3.00)	34.34 (2.24)	<0.001

Data are presented as *n* (%) or mean (SD). *p* values were derived from the chi-square test for categorical variables and the *t* test for continuous variables.

**Table 2:** Temporal trends in 1-year particulate matter exposure and HbA1c levels across study visits.

Visit	Year	*n*	PM_10_, 1-Year (μg/m^3^)	PM_2.5_, 1-Year (μg/m^3^)	HbA1c (%)
Wave 3	2005–2006	3275	66.86 (2.31)	33.21 (1.59)	5.37 (0.37)
Wave 4	2007–2008	5622	63.31 (3.70)	31.96 (3.48)	5.49 (0.41)
Wave 5	2009–2010	5605	55.14 (2.75)	26.58 (3.72)	5.61 (0.45)
Wave 6	2011–2012	5296	55.76 (5.17)	29.74 (3.86)	5.59 (0.48)
Wave 7	2013–2014	5033	52.80 (4.85)	28.43 (5.19)	5.61 (0.50)
Wave 8	2015–2016	5346	50.26 (3.57)	25.70 (4.02)	5.71 (0.56)
Wave 9	2017–2018	5218	50.56 (3.81)	25.16 (2.53)	5.76 (0.60)

Data are presented as mean (SD).

**Table 3:** Linear mixed-model estimates for associations between long-term PM exposure and HbA1c levels.

Variable	PM_10_	*p*	PM_2.5_	*p*
PM exposure (per IQR increase)	0.0347 (0.0220, 0.0473)	<0.001	0.0166 (0.0010, 0.0321)	0.037
Sex (female vs. male)	0.0638 (0.0397, 0.0878)	<0.001	0.0646 (0.0406, 0.0887)	<0.001
Education (reference: ≤middle school)				
High school	0.0140 (−0.0094, 0.0374)	0.241	0.0137 (−0.0098, 0.0371)	0.252
≥College	0.0219 (−0.0084, 0.0523)	0.157	0.0215 (−0.0089, 0.0519)	0.166
Region (Ansan vs. Ansung)	−0.0095 (−0.0315, 0.0125)	0.397	−0.0159 (−0.0395, 0.0077)	0.187
Age (per year)	0.0054 (0.0042, 0.0066)	<0.001	0.0053 (0.0041, 0.0066)	<0.001
BMI (per kg/m^2^)	0.0304 (0.0279, 0.0328)	<0.001	0.0304 (0.0279, 0.0328)	<0.001
Smoking status (reference: never)				
Former	0.0329 (0.0135, 0.0523)	<0.001	0.0331 (0.0137, 0.0525)	<0.001
Current	0.0589 (0.0356, 0.0822)	<0.001	0.0589 (0.0356, 0.0823)	<0.001
Alcohol consumption (reference: never)				
Former	0.0279 (0.0081, 0.0478)	0.006	0.0271 (0.0073, 0.0470)	0.007
Current	−0.0182 (−0.0311, −0.0054)	0.006	−0.0186 (−0.0315, −0.0058)	0.005
Regular exercise (yes vs. no)	−0.0140 (−0.0232, −0.0047)	<0.001	−0.0139 (−0.0231, −0.0046)	<0.001
Visit (reference: Wave 3)				
Wave 4	0.1245 (0.1087, 0.1402)	<0.001	0.1108 (0.0960, 0.1256)	<0.001
Wave 5	0.2653 (0.2425, 0.2882)	<0.001	0.2322 (0.2124, 0.2520)	<0.001
Wave 6	0.2256 (0.2028, 0.2485)	<0.001	0.1887 (0.1711, 0.2063)	<0.001
Wave 7	0.2539 (0.2272, 0.2805)	<0.001	0.2086 (0.1889, 0.2284)	<0.001
Wave 8	0.3461 (0.3160, 0.3763)	<0.001	0.2969 (0.2733, 0.3205)	<0.001
Wave 9	0.3869 (0.3560, 0.4178)	<0.001	0.3397 (0.3142, 0.3652)	<0.001

Values are presented as β (95% CI). β indicates the absolute change in HbA1c (percentage points) per unit increase in the listed predictor. PM_10_ IQR = 9.48 μg/m^3^; PM_2.5_ IQR = 8.67 μg/m^3^. Reference categories: male, ≤middle school, Ansung, never smoker, never drinker, no regular exercise, and Wave 3.

**Table 4:** Subgroup analysis of the association between long-term (1-year) PM exposure and HbA1c.

Subgroup	Category	No. of Subjects	No. of Observations	PM_10_			PM_2.5_		
				β (95% CI)	*p*	*p* for Interaction	β (95% CI)	*p*	*p* for Interaction
Overall		6940	35,395	0.0347 (0.0220, 0.0473)	<0.001		0.0166 (0.0010, 0.0321)	0.037	
Sex	Male	3250	16,471	0.0103 (−0.0093, 0.0299)	0.303	0.339	−0.0206 (−0.0444, 0.0033)	0.090	0.588
	Female	3690	18,924	0.0537 (0.0371, 0.0704)	<0.001		0.0474 (0.0269, 0.0679)	<0.001	
Age	<60 years	4394	23,456	0.0210 (0.0060, 0.0361)	0.006	<0.001	0.0024 (−0.0162, 0.0210)	0.800	<0.001
	≥60 years	2546	11,939	0.0789 (0.0549, 0.1028)	<0.001		0.0692 (0.0399, 0.0986)	<0.001	
BMI	<25 kg/m^2^	4246	21,506	0.0377 (0.0237, 0.0518)	<0.001	<0.001	0.0190 (0.0017, 0.0364)	0.032	<0.001
	≥25 kg/m^2^	2694	13,889	0.0264 (0.0021, 0.0507)	0.033		0.0027 (−0.0266, 0.0320)	0.857	
Region	Ansan	3435	17,779	0.0398 (0.0228, 0.0568)	<0.001		0.0265 (0.0033, 0.0497)	0.025	<0.001
	Ansung	3505	17,616	0.0963 (0.0733, 0.1194)	<0.001	<0.001	0.0894 (0.0575, 0.1212)	<0.001	
Education	≤Middle school	3744	18,588	0.0637 (0.0447, 0.0827)	<0.001	<0.001	0.0502 (0.0272, 0.0732)	<0.001	<0.001
	High school	2195	11,559	0.0259 (0.0059, 0.0459)	0.011		0.0041 (−0.0213, 0.0294)	0.751	
	≥College	1001	5248	0.0061 (−0.0282, 0.0405)	0.728		−0.0158 (−0.0598, 0.0282)	0.482	
Smoking	Never	4372	22,561	0.0455 (0.0298, 0.0613)	<0.001	0.035	0.0326 (0.0132, 0.0520)	<0.001	0.046
	Former	1306	6631	0.0043 (−0.0240, 0.0326)	0.766		−0.0207 (−0.0549, 0.0134)	0.235	
	Current	1262	6203	0.0256 (−0.0068, 0.0581)	0.122		−0.0052 (−0.0452, 0.0348)	0.799	
Drinking	Never	3341	16,927	0.0572 (0.0382, 0.0762)	<0.001	0.617	0.0438 (0.0209, 0.0667)	<0.001	0.917
	Former	334	1555	−0.0099 (−0.0797, 0.0599)	0.781		−0.0107 (−0.0968, 0.0755)	0.808	
	Current	3265	16,913	0.0168 (−0.0007, 0.0343)	0.060		−0.0079 (−0.0297, 0.0140)	0.479	
Exercise	No	4404	22,136	0.0385 (0.0211, 0.0559)	<0.001	0.364	0.0138 (−0.0073, 0.0350)	0.201	0.879
	Yes	2536	13,259	0.0319 (0.0135, 0.0502)	<0.001		0.0234 (0.0003, 0.0464)	0.047	

β, absolute change in HbA1c (percentage points) per IQR increase in PM_10_ (9.48 μg/m^3^) or PM_2.5_ (8.67 μg/m^3^); all models adjusted for sex, education, region, age, BMI, smoking status, drinking status, regular exercise, and study visit (the stratifying variable was excluded from each subgroup-specific model); *p* for interaction, Wald test *p* value for multiplicative interaction. BMI, body mass index; CI, confidence interval; IQR, interquartile range. All subgroup analyses were pre-specified.

## Data Availability

The data presented in this study are available from the Korean Genome and Epidemiology Study (KoGES; 4851-302), National Institute of Health, Korea Disease Control and Prevention Agency, Republic of Korea. Interested researchers may obtain the data from the NIH (https://nih.go.kr) upon reasonable request (accessed on 27 June 2023).

## Supplementary Materials

The following supporting information can be downloaded at: https://www.mdpi.com/article/10.3390/jcm15072797/s1, Table S1: Sensitivity analysis of PM–HbA1c associations across different exposure windows; Table S2: Multicollinearity diagnostics and correlation structure for the PM_10_ and PM_2.5_ 1-year exposure models.

## Abbreviations

The following abbreviations are used in this manuscript:

T2DM	Type 2 diabetes mellitus
PM_2.5_	Fine particulate matter
DALY	Disability-adjusted life-years
RR	Relative risk
CI	Confidence interval
HbA1c	Glycated hemoglobin
WHO	World Health Organization
KNHANES	Korea National Health and Nutrition Examination Survey
KoGES	Korean Genome and Epidemiology Study
PM	Particulate matter
BMI	Body mass index
PM_10_	Coarse particulate matter
CMAQ	Community Multiscale Air Quality
LMM	Linear mixed model
IQR	Interquartile range
SD	Standard deviation
HR	Hazard ratio

## References

[1] International Diabetes Federation (2021). IDF Diabetes Atlas.

[2] Zheng Y., Ley S.H., Hu F.B. (2018). Global aetiology and epidemiology of type 2 diabetes mellitus and its complications. Nat. Rev. Endocrinol..

[3] Balti E.V., Echouffo-Tcheugui J.B., Yako Y.Y., Kengne A.P. (2014). Air pollution and risk of type 2 diabetes mellitus: A systematic review and meta-analysis. Diabetes Res. Clin. Pract..

[4] Eze I.C., Hemkens L.G., Bucher H.C., Hoffmann B., Schindler C., Künzli N., Schikowski T., Probst-Hensch N.M. (2015). Association between ambient air pollution and diabetes mellitus in Europe and North America: Systematic review and meta-analysis. Environ. Health Perspect..

[5] Burkart K., Causey K., Cohen A.J., Wozniak S.S., Salvi D.D., Abbafati C., Adekanmbi V., Adsuar J.C., Ahmadi K., Alahdab F. (2022). Estimates, trends, and drivers of the global burden of type 2 diabetes attributable to PM_2.5_ air pollution, 1990–2019: An analysis of data from the Global Burden of Disease Study 2019. Lancet Planet. Health.

[6] Wu Y., Fu R., Lei C., Deng Y., Lou W., Wang L., Zheng Y., Deng X., Yang S., Wang M. (2021). Estimates of type 2 diabetes mellitus burden attributable to particulate matter pollution and its 30-year change patterns: A systematic analysis of data from the Global Burden of Disease Study 2019. Front. Endocrinol..

[7] Rajagopalan S., Brook R.D. (2012). Air pollution and type 2 diabetes: Mechanistic insights. Diabetes.

[8] Sun Q., Yue P., Deiuliis J.A., Lumeng C.N., Kampfrath T., Mikolaj M.B., Cai Y., Ostrowski M.C., Lu B., Parthasarathy S. (2009). Ambient air pollution exaggerates adipose inflammation and insulin resistance in a mouse model of diet-induced obesity. Circulation.

[9] Thangavel P., Park D., Lee Y.C. (2022). Recent insights into particulate matter (PM_2.5_)-mediated toxicity in humans: An overview. Int. J. Environ. Res. Public Health.

[10] Ying Z., Xu X., Bai Y., Zhong J., Chen M., Liang Y., Zhao J., Liu D., Morishita M., Sun Q. (2014). Long-term exposure to concentrated ambient PM_2.5_ increases mouse blood pressure through abnormal activation of the sympathetic nervous system: A role for hypothalamic inflammation. Environ. Health Perspect..

[11] Xu X., Liu C., Xu Z., Tzan K., Zhong M., Wang A., Lippmann M., Chen L.C., Rajagopalan S., Sun Q. (2011). Long-term exposure to ambient fine particulate pollution induces insulin resistance and mitochondrial alteration in adipose tissue. Toxicol. Sci..

[12] Liu C., Xu X., Bai Y., Wang T.Y., Rao X., Wang A., Sun L., Ying Z., Gushchina L., Maiseyeu A. (2014). Air pollution-mediated susceptibility to inflammation and insulin resistance: Influence of CCR2 pathways in mice. Environ. Health Perspect..

[13] He D., Wu S., Zhao H., Qiu H., Fu Y., Li X., He Y. (2017). Association between particulate matter 2.5 and diabetes mellitus: A meta-analysis of cohort studies. J. Diabetes Investig..

[14] Bowe B., Xie Y., Li T., Yan Y., Xian H., Al-Aly Z. (2018). The 2016 global and national burden of diabetes mellitus attributable to PM_2.5_ air pollution. Lancet Planet. Health.

[15] Chung W.S., Lin C.L. (2024). Exposure to fine particulate matter increases risk of diabetes mellitus: A population-based cohort study. J. Occup. Environ. Med..

[16] Shen Y., Jiang L., Xie X., Meng X., Xu X., Dong J., Yang Y., Xu J., Zhang Y., Wang Q. (2024). Long-term exposure to fine particulate matter and fasting blood glucose and diabetes in 20 million Chinese women of reproductive age. Diabetes Care.

[17] Kim H., Byun G., Choi Y., Kim S., Kim S.Y., Lee J.T. (2021). Effects of long-term exposure to air pollution on all-cause mortality and cause-specific mortality in seven major cities of South Korea: Korean national health and nutritional examination surveys with mortality follow-up. Environ. Res..

[18] Moon J., Kim E., Jang H., Song I., Kwon D., Kang C., Oh J., Park J., Kim A., Choi M. (2024). Long-term exposure to PM_2.5_ and mortality: A national health insurance cohort study. Int. J. Epidemiol..

[19] World Health Organization (2021). WHO Global Air Quality Guidelines: Particulate Matter (PM_2.5_ and PM_10_), Ozone, Nitrogen Dioxide, Sulfur Dioxide and Carbon Monoxide.

[20] Hwang M.J., Kim J.H., Koo Y.S., Yun H.Y., Cheong H.K. (2020). Impacts of ambient air pollution on glucose metabolism in Korean adults: A Korea National Health and Nutrition Examination Survey study. Environ. Health.

[21] Kim Y., Han B.G., KoGES Group (2017). Cohort Profile: The Korean Genome and Epidemiology Study (KoGES) Consortium. Int. J. Epidemiol..

[22] Woo H.D., Song D.S., Choi S.H., Park J.K., Lee K., Yun H.Y., Choi D.R., Koo Y.S., Park H.Y. (2022). Integrated dataset of the Korean Genome and Epidemiology Study cohort with estimated air pollution data. Epidemiol. Health.

[23] Kim B., Choi H.Y., Kim W., Ahn C., Lee J., Kim J.G., Kim J., Shin H., Yu J.M., Moon S. (2018). The cut-off values of surrogate measures for insulin resistance in the Korean population according to the Korean Genome and Epidemiology Study (KOGES). PLoS ONE.

[24] Bio-Rad Laboratories VARIANT II Hemoglobin Testing System. https://www.bio-rad.com/en-us/product/variant-ii-hemoglobin-testing-system?ID=49373a90-6f08-4684-a7d8-8afd7e0564ef.

[25] American Diabetes Association Professional Practice Committee (2024). 2. Diagnosis and Classification of Diabetes: Standards of Care in Diabetes-2024. Diabetes Care.

[26] Honda T., Pun V.C., Manjourides J., Suh H. (2017). Associations between long-term exposure to air pollution, glycosylated hemoglobin and diabetes. Int. J. Hyg. Environ. Health.

[27] Lucht S.A., Hennig F., Matthiessen C., Ohlwein S., Icks A., Moebus S., Jöckel K.H., Jakobs H., Hoffmann B. (2018). Air pollution and glucose metabolism: An analysis in non-diabetic participants of the Heinz Nixdorf Recall Study. Environ. Health Perspect..

[28] Rose G. (1985). Sick individuals and sick populations. Int. J. Epidemiol..

[29] Li Y., Wu J., Tang H., Jia X., Wang J., Meng C., Wang W., Liu S., Yuan H., Cai J. (2024). Long-term PM_2.5_ exposure and early-onset diabetes: Does BMI link this risk?. Sci. Total Environ..

[30] Furukawa S., Fujita T., Shimabukuro M., Iwaki M., Yamada Y., Nakajima Y., Nakayama O., Makishima M., Matsuda M., Shimomura I. (2004). Increased oxidative stress in obesity and its impact on metabolic syndrome. J. Clin. Investig..

[31] Kahn B.B., Flier J.S. (2000). Obesity and insulin resistance. J. Clin. Investig..

[32] Chen H., Burnett R.T., Kwong J.C., Villeneuve P.J., Goldberg M.S., Brook R.D., van Donkelaar A., Jerrett M., Martin R.V., Brook J.R. (2013). Risk of incident diabetes in relation to long-term exposure to fine particulate matter in Ontario, Canada. Environ. Health Perspect..

[33] Liang F., Yang X., Liu F., Li J., Xiao Q., Chen J., Liu X., Cao J., Shen C., Yu L. (2019). Long-term exposure to ambient fine particulate matter and incidence of diabetes in China: A cohort study. Environ. Int..

[34] Li S., Guo B., Jiang Y., Wang X., Chen L., Wang X., Chen T., Yang L., Silang Y., Hong F. (2023). Long-term exposure to ambient PM_2.5_ and its components associated with diabetes: Evidence from a large population-based cohort from China. Diabetes Care.

[35] Finkel T., Holbrook N.J. (2000). Oxidants, oxidative stress and the biology of ageing. Nature.

[36] Shumake K.L., Sacks J.D., Lee J.S., Johns D.O. (2013). Susceptibility of older adults to health effects induced by ambient air pollutants regulated by the European Union and the United States. Aging Clin. Exp. Res..

[37] Liu X., Jiang N., Yu X., Zhang R., Li S., Li Q., Kang P. (2019). Chemical characteristics, sources apportionment, and risk assessment of PM2.5 in different functional areas of an emerging megacity in China. Aerosol Air Qual. Res..

[38] Cai C., Zhu S., Qin M., Li X., Feng C., Yu B., Dai S., Qiu G., Li Y., Ye T. (2024). Long-term exposure to PM_2.5_ chemical constituents and diabesity: Evidence from a multi-center cohort study in China. Lancet Reg. Health West. Pac..

[39] Liu T., Meng H., Yu M., Xiao Y., Huang B., Lin L., Zhang H., Hu R., Hou Z., Xu Y. (2021). Urban-rural disparity of the short-term association of PM_2.5_ with mortality and its attributable burden. Innovation.

[40] O’Neill M.S., Jerrett M., Kawachi I., Levy J.I., Cohen A.J., Gouveia N., Wilkinson P., Fletcher T., Cifuentes L., Schwartz J. (2003). Health, wealth, and air pollution: Advancing theory and methods. Environ. Health Perspect..

[41] Hajat A., Hsia C., O’Neill M.S. (2015). Socioeconomic disparities and air pollution exposure: A global review. Curr. Environ. Health Rep..

[42] Gee G.C., Payne-Sturges D.C. (2004). Environmental health disparities: A framework integrating psychosocial and environmental concepts. Environ. Health Perspect..

[43] Andersen Z.J., Raaschou-Nielsen O., Ketzel M., Jensen S.S., Hvidberg M., Loft S., Tjønneland A., Overvad K., Sørensen M. (2012). Diabetes incidence and long-term exposure to air pollution: A cohort study. Diabetes Care.

[44] Niemann B., Rohrbach S., Miller M.R., Newby D.E., Fuster V., Kovacic J.C. (2017). Oxidative stress and cardiovascular risk: Obesity, diabetes, smoking, and pollution: Part 3 of a 3-part series. J. Am. Coll. Cardiol..

[45] Yitshak Sade M., Kloog I., Liberty I.F., Schwartz J., Novack V. (2016). The association between air pollution exposure and glucose and lipids levels. J. Clin. Endocrinol. Metab..

[46] Pope C.A., Burnett R.T., Thurston G.D., Thun M.J., Calle E.E., Krewski D., Godleski J.J. (2004). Cardiovascular mortality and long-term exposure to particulate air pollution: Epidemiological evidence of general pathophysiological pathways of disease. Circulation.

[47] Brook R.D., Rajagopalan S., Pope C.A., Brook J.R., Bhatnagar A., Diez-Roux A.V., Holguin F., Hong Y., Luepker R.V., Mittleman M.A. (2010). Particulate matter air pollution and cardiovascular disease: An update to the scientific statement from the American Heart Association. Circulation.

[48] Xu J., Zhang W., Lu Z., Zhang F., Ding W. (2017). Airborne PM_2.5_-induced hepatic insulin resistance by Nrf2/JNK-mediated signaling pathway. Int. J. Environ. Res. Public Health.

[49] Lu Y., Qiu W., Liao R., Cao W., Huang F., Wang X., Li M., Li Y. (2025). Subacute PM2.5 exposure induces hepatic insulin resistance through inflammation and oxidative stress. Int. J. Mol. Sci..

