# Supplementary material for: Association Between Long-Term Exposure to Particulate Matter and Glycated Hemoglobin Levels: A Cohort Study from the Korean Genome and Epidemiology Study
Source: J Clin Med. 2026 Apr 7;15(7):2797. doi: 10.3390/jcm15072797 (PMC13074078; doi:10.3390/jcm15072797)
Supplement: Supplementary file 1 [file jcm-15-02797-s001.zip › jcm-4181739-supplementary.pdf]

Table S1. Sensitivity analysis of the association between PM exposure and HbA1c across different exposure time windows.

Exposure Window	IQR (μg/m³)	β	95% CI	p-value
**PM_{10}**				
1-week	24.69	−0.0094	−0.0147, −0.0042	<0.001
1-month	22.89	−0.0022	−0.0089, 0.0046	0.526
3-month	18.75	0.0074	0.0005, 0.0143	0.036
6-month	12.97	0.0056	−0.0018, 0.0129	0.137
1-year	9.48	0.0347	0.0220, 0.0473	<0.001
**$PM_{2.5}$**				
1-week	13.59	−0.0130	−0.0186, −0.0074	<0.001
1-month	11.85	−0.0197	−0.0267, −0.0126	<0.001
3-month	10.52	−0.0094	−0.0177, −0.0012	0.025
6-month	9.54	−0.0118	−0.0218, −0.0017	0.022
1-year	8.67	0.0166	0.0010, 0.0321	0.037

β, absolute change in HbA1c (percentage points) per interquartile range (IQR) increase in PM concentration. All models were adjusted for sex, age, body mass index, educational attainment, region, smoking status, drinking status, regular exercise, and study visit. N = 35,395 observations from 6,940 participants. Observation counts are slightly lower than the primary analysis (N = 35,404) due to missing PM estimates for some exposure windows.

Table S2. Multicollinearity diagnostics and correlation structure for the PM_{10} and $PM_{2.5}$ 1-year exposure models.

(a) Generalized variance inflation factors (GVIF)

Variable	PM_{10}		$PM_{2.5}$	
	GVIF	Adjusted GVIF	GVIF	Adjusted GVIF
PM exposure (per IQR)	5.044	2.246	4.826	2.197
Sex	2.646	1.627	2.644	1.626
Educational attainment	1.464	1.100	1.465	1.100
Region	2.014	1.419	3.423	1.850
Age	1.589	1.261	1.589	1.261
BMI	1.023	1.012	1.023	1.012
Smoking status	2.493	1.256	2.493	1.257
Drinking status	1.549	1.116	1.549	1.116
Regular exercise	1.093	1.045	1.093	1.045
Study visit (Wave 3–9)	4.732	1.138	3.108	1.099

GVIF = generalized variance inflation factor; Adjusted GVIF = GVIF^(1/(2×Df)), where Df is the degrees of freedom for each variable, which accounts for multi-category variables and is comparable to the square ro ot of VIF for single-degree-of-freedom terms. Values < 2.5 indicate acceptable multicollinearity.

(b) Pearson correlation matrix

Variable	Visit number	PM_{10}	$PM_{2.5}$	HbA1c
Visit number	1.000	−0.755	−0.499	0.207
PM_{10}	−0.755	1.000	0.855	−0.137
$PM_{2.5}$	−0.499	0.855	1.000	−0.077
HbA1c	0.207	−0.137	−0.077	1.000

All correlations were statistically significant ($p < 0.001$). N = 35,395 observations from 6,940 participants.
